# Optimizing the Management of Pediatric Bronchiolitis: A Multi-cycle Clinical Audit at a Regional Hospital in Oman

**DOI:** 10.7759/cureus.104761

**Published:** 2026-03-06

**Authors:** Latifa Al Maamari, Nasser Al Shafouri, Mahmoud Khaild, Ekram Elsiddig, Amna Alghassani

**Affiliations:** 1 Pediatrics and Neonatology, Ibri Hospital, Ibri, OMN; 2 Pharmacy, Ibri Hospital, Ibri, OMN

**Keywords:** bronchiolitis, clinical audit, guideline adherence, medication stewardship, quality improvement, supportive care, unnecessary investigations

## Abstract

Background

Bronchiolitis is a major cause of lower respiratory tract infection and hospital admission among infants. Although international guidelines consistently recommend supportive care as the primary management approach, significant variation in clinical practice persists, often resulting in avoidable investigations, treatments, and healthcare expenditure, contributing to unnecessary costs and potential patient harm from avoidable interventions.

Objective

The objective of this study is to evaluate the effectiveness of a multi-cycle clinical audit in improving adherence to evidence-based bronchiolitis management guidelines at a regional hospital in Oman.

Methods

A sequential four-cycle clinical audit was carried out at Ibri Regional Hospital between March 2022 and October 2024. Audit standards were based on the 2014 American Academy of Pediatrics (AAP) Clinical Practice Guideline for the diagnosis, management, and prevention of bronchiolitis. Following each cycle, targeted educational and feedback interventions were introduced, and subsequent changes in clinical practice were evaluated. Data were retrospectively extracted from electronic medical records (EMR) and assessed for trends in improvement across audit cycles.

Results

Across 210 admissions, the median patient age was six months (interquartile range: 3-10 months), and 85% were under 12 months of age. Over the four cycles, chest radiography (CXR) decreased from 82% (18/22) to 40.9% (27/66) (p<0.001), salbutamol use decreased from 91% (20/22) to 22.7% (15/66) (p<0.001), ipratropium bromide use decreased from 59% (13/22) to 9% (6/66) (p<0.001), corticosteroid use decreased from 31% (7/22) to 6.06% (4/66) (p<0.001), and antibiotic prescriptions decreased from 45% (10/22) to 15.1% (10/66) (p<0.001). Supportive care improved markedly: Intravenous (IV) fluids increased from 50% (11/22) to 92.4% (61/66) (p<0.001), and nasopharyngeal suctioning increased from 18% (4/22) to 98.4% (65/66) (p<0.001).

Conclusion

Sequential audit cycles with targeted educational interventions significantly improved adherence to evidence-based bronchiolitis management. This approach offers a feasible, replicable model for improving pediatric respiratory care in regional hospital settings.

## Introduction

Acute bronchiolitis is the most common lower respiratory tract infection in infants, accounting for over 130,000 hospitalizations annually in the United States, with direct hospital costs exceeding $700 million [1]. Despite its prevalence and the existence of clear evidence-based guidelines from major pediatric organizations, considerable practice variation persists globally in the management of this self-limiting viral illness [2].

The pathophysiology of bronchiolitis involves the virus-induced inflammation of the bronchioles, leading to edema, increased mucus production, and bronchiolar obstruction. Respiratory syncytial virus (RSV) accounts for 50%-80% of cases, with other viruses, including rhinovirus, parainfluenza, and human metapneumovirus, contributing to the remaining cases [3]. The disease typically affects children under 24 months of age, with peak incidence occurring between three and six months of age [3].

International guidelines consistently recommend supportive care as the cornerstone of management. The American Academy of Pediatrics (AAP), National Institute for Health and Care Excellence (NICE), and Canadian Paediatric Society explicitly discourage the routine use of diagnostic tests, bronchodilators, corticosteroids, and antibiotics, as robust evidence demonstrates their lack of efficacy in altering disease course or improving clinically meaningful outcomes [4].

Despite this evidence, worldwide studies continue to document the overuse of ineffective interventions. A systematic review found that chest radiography was performed in 46%-69% of bronchiolitis cases, antibiotics were prescribed in 24%-38%, and bronchodilators were administered in 48%-78% of the patients [5]. This practice variation not only increases healthcare costs but also may expose infants to unnecessary risks without providing clinical benefit.

The Middle Eastern region, including Oman, faces unique challenges in implementing evidence-based bronchiolitis management. Factors including high parental expectations, physician concerns about missing bacterial infections, and the limited awareness of current guidelines contribute to overtreatment. Previous studies from Gulf Cooperation Council (GCC) countries have reported similar patterns of non-adherence to international guidelines, highlighting the need for targeted quality improvement initiatives [6].

Clinical audit represents a powerful quality improvement tool for bridging the evidence-practice gap. Sequential audit cycles with Plan-Do-Study-Act (PDSA) methodology have demonstrated effectiveness in changing physician behavior and improving guideline adherence across various clinical conditions [7]. The audit cycle involves systematically reviewing current practice against established standards, implementing targeted interventions, and reassessing to measure improvement.

This study aimed to evaluate the effectiveness of a four-cycle sequential clinical audit in improving adherence to AAP 2014 evidence-based bronchiolitis management guidelines at Ibri Regional Hospital, Oman, specifically targeting the reduction of unnecessary investigations and ineffective treatments while improving appropriate supportive care delivery. We hypothesized that sequential audit cycles with targeted educational interventions would significantly reduce unnecessary investigations and ineffective treatments while improving supportive care measures.

## Materials and methods

This quality improvement study was conducted at Ibri Regional Hospital, a secondary care facility serving the Al Dhahirah Governorate in Oman. The hospital operates a 29-bed pediatric ward that manages approximately 200 cases of acute bronchiolitis annually. The study utilized a sequential audit methodology comprising four complete audit cycles conducted over a 31-month period from March 2022 to October 2024. This timeframe allowed sufficient intervals between cycles for the implementation of educational interventions and the assessment of their sustained impact on clinical practice patterns.

Case definition and study population

The study population consisted of all children admitted to the pediatric ward with a primary diagnosis of acute bronchiolitis during the designated study periods. The American Academy of Pediatrics (AAP) diagnostic criteria were employed, defining acute bronchiolitis as the first episode of wheezing in children under 24 months of age that was preceded by symptoms of upper respiratory tract infection [4]. No patients were excluded from the analysis regardless of disease severity, comorbidities, or management approach.

The baseline audit (Cycle 1) was conducted from March to May 2022 and included 22 patients (n=22). The first re-audit (Cycle 2) involved 53 patients (n=53) over a two-month period from September to October 2022. The second re-audit (Cycle 3) evaluated 69 patients (n=69) admitted between October and December 2023, while the third re-audit (Cycle 4) analyzed 66 patients (n=66) from July to October 2024.

Audit standards and benchmarks

Audit standards were derived from the 2014 American Academy of Pediatrics (AAP) Clinical Practice Guideline for the diagnosis, management, and prevention of bronchiolitis, supplemented by adapted local protocols based on Royal Hospital Oman institutional guidelines [4].

For diagnostic evaluation, the standards specified that diagnosis should be established through comprehensive history-taking and physical examination alone, without routine radiographic or laboratory investigations. Chest radiography (CXR) was considered acceptable only in cases of atypical clinical presentations, for children with an age of less than six months or greater than 18 months, or when complications such as pneumothorax or lobar consolidation were clinically suspected. Similarly, laboratory investigations, including C-reactive protein (CRP) measurement and blood cultures, were not recommended for routine use in uncomplicated bronchiolitis.

Regarding therapeutic management, the standards explicitly discouraged the use of bronchodilators, including both salbutamol and ipratropium bromide, as evidence demonstrates no clinically meaningful benefit in altering the disease pathway or improving patient outcomes. Systemic corticosteroids were similarly contraindicated in all settings due to the lack of efficacy and potential adverse effects. Antibiotic prescription was considered inappropriate except in cases with documented bacterial co-infection, such as otitis media, bacterial pneumonia, or urinary tract infection.

The cornerstone of appropriate management emphasized supportive care measures, including adequate hydration through intravenous (IV) fluid administration when oral intake proved insufficient, supplemental oxygen therapy for infants with oxygen saturation below 90%, and regular nasopharyngeal suctioning to maintain airway patency and reduce respiratory effort.

Data collection methodology

Data were retrospectively extracted from the AlShifa 3plus electronic medical record (EMR) system for all patients included in each audit cycle. The data collection process was standardized across all four cycles to ensure consistency and comparability.

Demographic variables extracted included patient age at presentation, gender, and documented comorbid conditions such as prematurity, congenital heart disease, chronic lung disease, or immunodeficiency. Clinical presentation parameters encompassed the presence and duration of fever, the severity of respiratory distress, and documented vital signs.

Investigation-related variables captured the ordering and results of chest radiography (CXR), C-reactive protein (CRP) levels, blood culture specimens, and respiratory viral panel (RVP) testing when performed. Treatment variables comprehensively documented all nebulized therapies administered, including salbutamol, ipratropium bromide, normal saline, 3% hypertonic saline, and nebulized adrenaline. Systemic medication administration was recorded for antibiotics (with specific agents and stated indications), corticosteroids (including dexamethasone, hydrocortisone, and prednisolone), and antiviral agents such as oseltamivir.

Supportive care measures documented included intravenous (IV) fluid therapy, supplemental oxygen delivery methods, and nasopharyngeal suctioning. Advanced respiratory support modalities were noted when employed, specifically high-flow nasal cannula oxygen therapy and continuous positive airway pressure (CPAP) ventilation. Finally, discharge diagnosis accuracy was assessed by comparing initial working diagnoses with final documented diagnoses to identify cases where alternative conditions were ultimately diagnosed.

Quality improvement intervention strategy

Following the completion of each audit cycle, a comprehensive intervention strategy was implemented to address identified deficiencies and reinforce evidence-based practices. The results from each cycle were systematically analyzed and shared with all pediatric and emergency department staff members, including consultant pediatricians, specialized pediatricians, resident physicians, and nursing staff.

Educational interventions took multiple forms to maximize impact across different learning preferences and clinical contexts. Interactive educational sessions were conducted in the form of departmental lectures and case-based discussions that highlighted specific guideline recommendations while contextualizing them within the audit findings from our institution. These sessions emphasized the evidence base supporting each recommendation and addressed common misconceptions about bronchiolitis management.

Visual reminder systems were established through strategically placed posters displaying the American Academy of Pediatrics (AAP) guideline algorithm in high-traffic clinical areas, including the emergency department triage area, pediatric ward nursing station, and resident physicians' workrooms. Individualized feedback was provided through direct discussions with physicians whose documented practice patterns deviated substantially from established guidelines, ensuring these conversations remained educational and nonpunitive in nature.

Protocol reinforcement occurred through the distribution of simplified, laminated management protocols that could be easily referenced at the point of care. Recognizing that many management decisions were initiated in the emergency department before pediatric consultation, dedicated collaborative sessions were organized with emergency medicine physicians and nursing staff to align initial assessment and management approaches with evidence-based guidelines.

The main form of resistance encountered during feedback sessions was physician concern regarding missing bacterial co-infections, which likely contributed to the persistent use of CRP testing, blood cultures, chest radiography, and antibiotic prescribing observed in later cycles. This was addressed through structured case discussions emphasizing the clinical criteria distinguishing viral bronchiolitis from bacterial infection, the low prevalence of bacterial co-infection in this age group, and the importance of clinical reassessment over routine investigation.

Statistical analysis

Data were analyzed using SPSS version 26.0 (IBM Corp., Armonk, NY). Continuous variables were presented as medians with interquartile ranges, while categorical variables were expressed as frequencies and percentages. Chi-square tests for trend assessed the statistical significance of changes in proportions across audit cycles, with Fisher's exact test applied when cell counts were insufficient. Statistical significance was defined as a two-tailed p-value of less than 0.05. Trend analysis evaluated progressive improvement across the four cycles to distinguish sustained practice change from transient intervention effects.

Ethical considerations

This study was conducted as a quality improvement project in accordance with the institutional guidelines of Ibri Regional Hospital. As the work constituted an audit of routine clinical care utilizing retrospectively collected data from existing medical records without patient identifiers, institutional approval was obtained. Data confidentiality was preserved with all analyses conducted on de-identified datasets. Access to medical records was limited to members of the clinical team directly involved in patient care and quality improvement activities. No additional interventions, investigations, or treatments beyond routine clinical care were performed for purposes of this audit.

## Results

Demographic characteristics

Across the four audit cycles, a total of 210 children were included in the analysis. The median age at presentation was six months (interquartile range: 3-10 months), with 85% (179/210) of the patients younger than 12 months of age. There was a male predominance, with a male-to-female ratio of 1.4:1. Respiratory syncytial virus (RSV) testing was performed inconsistently between cycles, with positivity rates ranging from 60% to 75% when tested. Common comorbidities among admitted children included prematurity (8%, 17/210), congenital heart disease (4%, 8/210), and chronic lung disease (3%, 6/210).

Diagnostic testing utilization

Progressive improvements were observed across most audit indicators. The use of chest radiography (CXR) declined steadily from 82% (18/22) in the first cycle to 40.9% (27/66) by the fourth cycle, representing a 50% relative reduction (p<0.001). A brief increase during the second cycle to 88.6% (47/53) likely reflected ongoing documentation of pre-intervention practices before the educational measures took effect.

Testing for C-reactive protein (CRP) initially showed modest change, remaining around 78% (17/22) in the first cycle and 77.4% (41/53) in the second cycle before declining to approximately 59.1% (41/69) in the third cycle and 59% (39/66) in the fourth cycle (p=0.0002). Blood culture requests showed a more dramatic decline, from 40% (9/22) at baseline to 17.3% (12/69) in the third cycle, followed by a slight rise to 31.8% (21/66) in the final cycle (p=0.0025 for overall trend). A review of these cases indicated that most cultures were ordered in the emergency department prior to the confirmation of a bronchiolitis diagnosis (Table 1).

**Table 1:** Diagnostic Test Utilization Across Audit Cycles Significant reductions in chest radiography (CXR) (82%-40.9%) and blood culture ordering. C-reactive protein usage declined modestly from 78% to 59%. Values presented as percentage (N) CRP: C-reactive protein

Diagnostic Test	Cycle 1 (n=22)	Cycle 2 (n=53)	Cycle 3 (n=69)	Cycle 4 (n=66)	P-value (Trend)
CXR Usage	82% (18)	88.6% (47)	55.1% (38)	40.9% (27)	<0.0001
CRP Usage	78% (17)	77.4% (41)	59.1% (41)	59% (39)	0.0002
Blood Culture	40% (9)	55% (29)	17.3% (12)	31.8% (21)	0.0025

Nebulized therapy utilization

Substantial gains were achieved in reducing the use of unnecessary nebulized therapies. Salbutamol administration fell from 91% (20/22) in the first cycle to 22.7% (15/66) in the final cycle, representing a 75% relative reduction (p<0.001), with most remaining use occurring in the emergency department before pediatric assessment. Ipratropium bromide use decreased from 59% (13/22) to 9% (6/66) over the same period, representing an 85% relative reduction (p<0.001).

In contrast, normal saline nebulization remained consistently used in 95% (21/22) to 100% (66/66) of cases, reflecting its accepted role in airway humidification and secretion clearance. Hypertonic saline (3% sodium chloride) was briefly introduced during the second cycle, reaching 9.4% (5/53) utilization before being discontinued in alignment with updated recommendations. Adrenaline use remained below 10% across all cycles and was restricted to more severe presentations (Table 2).

**Table 2:** Nebulized Treatment Modalities Across Audit Cycles Salbutamol decreased by 75% (91%-22.7%), and ipratropium decreased by 85% (59%-9%). Normal saline remained consistently high (95%-100%), while hypertonic saline was briefly introduced and then discontinued. Values presented as percentage (N) NaCl: sodium chloride

Nebulizer Type	Cycle 1 (n=22)	Cycle 2 (n=53)	Cycle 3 (n=69)	Cycle 4 (n=66)	P-value (Trend)
Salbutamol	91% (20)	56.6% (30)	24.6% (17)	22.7% (15)	<0.0001
Ipratropium	59% (13)	35.6% (19)	13% (9)	9% (6)	<0.0001
Normal Saline	95% (21)	96% (51)	97.1% (67)	100% (66)	0.24
3% NaCl	0% (0)	9.4% (5)	0% (0)	0% (0)	0.18
Adrenaline	0% (0)	9.4% (5)	2.9% (2)	3% (2)	0.31

Systemic medication and supportive care management

Patterns in systemic medication use improved considerably across the audit period. Antibiotic prescriptions fell from 45% (10/22) in the first cycle to 15.1% (10/66) in the fourth cycle, amounting to a 66% relative reduction (p=0.0015). Most antibiotic use in the later cycles was clinically justified, prescribed for confirmed bacterial co-infections such as otitis media, tonsillitis, or aspiration pneumonia. Nonetheless, some prescriptions were issued in response to prolonged fever or elevated inflammatory markers without confirmed bacterial infection.

Corticosteroid use decreased from 31% (7/22) to 6.06% (4/66), an 80% relative reduction (p=0.0001), with remaining cases largely reflecting diagnostic uncertainty between bronchiolitis and early-onset asthma. The use of oseltamivir (Tamiflu) fluctuated across the audit period, from 14% (3/22) to 28.3% (15/53), then 20.2% (14/69), and finally 31.8% (21/66), likely reflecting seasonal variation and increased influenza awareness during and after the coronavirus disease 2019 (COVID-19) pandemic.

Supportive management improved most strikingly. Intravenous (IV) fluid therapy rose from 50% (11/22) in the baseline cycle to 92.4% (61/66) in subsequent cycles (p=0.0001), reflecting better attention to hydration in infants with poor oral intake. Nasopharyngeal suctioning demonstrated the greatest increase, from 18% (4/22) at baseline to 98.4% (65/66) by the fourth cycle (p<0.0001), marking the successful integration of an evidence-based supportive intervention.

Oxygen therapy remained appropriately titrated to clinical severity, used in 18% (4/22) to 41.5% (22/53) of cases without a statistically significant trend (p=0.08), while advanced respiratory support, high-flow nasal cannula or continuous positive airway pressure (CPAP), was required in only 8%-15% of admissions and reserved for severe hypoxemia. Magnesium sulfate use remained minimal, ranging from 0% to 5% (1/22) across cycles (Table 3).

**Table 3:** Supportive Care and Systemic Medications Across Audit Cycles Intravenous (IV) fluids increased from 50% to 92.4%, and nasopharyngeal suctioning increased from 18% to 98.4%. Corticosteroids decreased by 80% (31%-6.06%), and antibiotics decreased by 66% (45%-15.1%). Values presented as percentage (N)

Management Type	Cycle 1 (n=22)	Cycle 2 (n=53)	Cycle 3 (n=69)	Cycle 4 (n=66)	P-value (Trend)
IV Fluids	50% (11)	98.1% (52)	92.7% (64)	92.4% (61)	0.0001
Nasopharyngeal Suction	18% (4)	73.5% (39)	89.8% (62)	98.4% (65)	<0.0001
Oxygen Supplementation	18% (4)	41.5% (22)	28.9% (20)	27.2% (18)	0.08
Corticosteroids	31% (7)	11.3% (6)	7.2% (5)	6.06% (4)	0.0001
Antibiotics	45% (10)	20% (11)	10.1% (7)	15.1% (10)	0.0015
Magnesium Sulfate	5% (1)	0% (0)	1.4% (1)	0% (0)	0.42
Oseltamivir (Tamiflu)	14% (3)	28.3% (15)	20.2% (14)	31.8% (21)	0.24

Diagnostic coding accuracy

Despite clear improvement in clinical practice, diagnostic coding inaccuracies persisted throughout the audit period. Several cases initially managed as bronchiolitis were later reassigned diagnoses such as asthma, bacterial pneumonia, or pertussis yet retained the bronchiolitis discharge code. This inconsistency, despite repeated educational sessions, underscored the ongoing need to strengthen electronic medical record systems to ensure accurate diagnosis reconciliation at discharge.

## Discussion

Overview of findings

This quality improvement initiative demonstrates that sequential clinical audit cycles with targeted educational interventions can substantially improve adherence to evidence-based bronchiolitis management guidelines in a secondary regional hospital setting. Over a 31-month period, the study achieved significant reductions in unnecessary investigations (50% reduction in chest radiography) and ineffective medications (75% reduction in salbutamol, 80% reduction in corticosteroids, and 66% reduction in antibiotics) and simultaneously increased implementation of appropriate supportive care measures (92.4% utilization of intravenous fluids and 98.4% nasopharyngeal suctioning).

Diagnostic investigations: Bridging the evidence-practice gap

The progressive reduction in chest radiography from 82% (18/22) to 40.9% (27/66) across audit cycles represents substantial improvement toward guideline adherence, though it remains above recommended standards. This pattern mirrors findings in other quality improvement initiatives targeting radiography overuse in pediatric respiratory infections [8]. The transient increase during Cycle 2 to 88.6% (47/53) likely reflects the expected "documentation effect" where initial audits identify preexisting practices before interventions take effect, a phenomenon well-described in audit methodology literature [8,9].

C-reactive protein (CRP) testing demonstrated more resistant change patterns, decreasing modestly from 78% (17/22) to 59% (39/66) by Cycles 3-4. This persistence may reflect deep-rooted clinical beliefs about inflammatory markers as disease severity indicators, despite robust evidence demonstrating their lack of clinical utility in bronchiolitis management [10]. Our findings align with systematic reviews documenting the difficulty in changing physician reliance on laboratory investigations, even when evidence clearly contradicts their use [11]. Recent literature continues to support that inflammatory markers lack predictive value for disease severity or clinical deterioration in viral bronchiolitis and contribute to inappropriate antibiotic prescribing [12].

Blood culture ordering showed dramatic improvement, declining from 40% (9/22) in the baseline cycle to 17.3% (12/69) in Cycle 3, though increasing again to 31.8% (21/66) in Cycle 4. This finding underscores the critical importance of targeting educational interventions at all care interfaces, particularly emergency departments that frequently serve as the initial assessment point for acute respiratory infections. Similar organizational challenges in guideline implementation have been identified in pediatric emergency medicine settings globally [13].

Nebulized therapy reduction: Addressing medication overuse

Salbutamol utilization decreased 75% across the study period, progressing from 91% (20/22) in Cycle 1 to 22.7% (15/66) in Cycle 4. This substantial reduction aligns with evidence from multiple randomized controlled trials demonstrating a lack of clinically meaningful benefit for bronchodilators in uncomplicated bronchiolitis [4,14]. Our findings are particularly noteworthy given that salbutamol use has remained persistently high in many healthcare systems despite decades of negative evidence, suggesting that our multi-faceted intervention approach proved more effective than educational efforts alone.

The Cochrane systematic review on bronchodilators for bronchiolitis, which analyzed 29 randomized controlled trials involving over 1,600 infants, concluded that bronchodilators offer no consistent or clinically meaningful improvement in clinical outcomes, supporting the reduction observed in our study [14]. The residual 22.7% (15/66) salbutamol use in Cycle 4 predominantly occurred in the emergency department prior to admission, a pattern consistent with published data demonstrating that initial management often reflects reactive prescribing rather than evidence-based protocols [2].

Ipratropium bromide use declined by 85%, from 59% (13/22) to 9% (6/66), demonstrating that anticholinergic agents, often prescribed as second-line agents after bronchodilators prove ineffective, respond readily to targeted intervention. Normal saline nebulization was appropriately maintained at high utilization rates (95%-100%), consistent with its evidence-supported role in secretion mobilization and humidification [15]. The brief introduction and subsequent discontinuation of 3% hypertonic saline demonstrate responsiveness to guideline updates and the appropriate adaptation of protocols based on evidence review [16].

Systemic medication management: Antibiotic stewardship

Antibiotic prescription rates decreased by 66%, from 45% (10/22) in Cycle 1 to 15.1% (10/66) in Cycle 4. This substantial improvement contributes meaningfully to antimicrobial stewardship efforts, a globally critical priority given increasing antimicrobial resistance [17]. Our findings demonstrate that quality improvement initiatives targeting unnecessary antibiotic use in viral respiratory infections can achieve clinically significant results comparable to dedicated stewardship programs [18].

The World Health Organization has emphasized that unnecessary antibiotic use in viral respiratory tract infections contributes significantly to the development of antimicrobial resistance [19]. Detailed case review in Cycles 3-4 revealed that most remaining antibiotic prescriptions appeared clinically justified for documented bacterial co-infections. However, some instances of persistent prescribing in the setting of isolated fever elevation or elevated inflammatory markers without an identified bacterial source suggest continued need for targeted education on the distinction between systemic inflammatory markers and confirmed bacterial infection [20].

Corticosteroid utilization declined by 80%, from 31% (7/22) to 6.06% (4/66), approaching international guideline recommendations for these agents. The remaining prescriptions occurred predominantly in children with a family history of asthma or recurrent wheezing, suggesting possible diagnostic uncertainty between acute viral bronchiolitis and asthma. Cochrane reviews and multiple clinical trials have demonstrated that systemic corticosteroids do not improve outcomes in uncomplicated bronchiolitis and should not be routinely used [21].

Supportive care enhancement: The most positive finding

The most impressive improvements were observed in supportive care implementation. Intravenous (IV) fluid therapy increased from 50% (11/22) to 92.4% (61/66), while nasopharyngeal suctioning increased from 18% (4/22) to 98.4% (65/66). These dramatic improvements represent successful behavioral change toward evidence-based practice for care measures that directly benefit patients.

The near-universal adoption of nasopharyngeal suctioning is particularly noteworthy, as this intervention requires nursing staff engagement and represents a fundamental shift from medication-focused to nursing-care-focused management philosophy. Evidence-based practice guidelines emphasize that nasopharyngeal suctioning with appropriate secretion removal is essential for improving airway patency and reducing the work of breathing in infants with bronchiolitis [4]. The 80-fold increase in suctioning utilization demonstrates that systematic quality improvement approaches can successfully change established nursing practices.

Adequate hydration through intravenous fluid administration is a cornerstone of supportive care in bronchiolitis, particularly in infants with severe respiratory distress who may be unable to tolerate oral intake [4]. The increase from 50% (11/22) to 92.4% (61/66) reflects the improved recognition of this fundamental management principle among clinical staff. Oxygen supplementation rates appear clinically appropriate and remained stable, suggesting that providers already applied reasonable judgment in oxygen titration to oxygen saturation targets.

Implementation science and quality improvement methodology

Several factors likely contributed to the success of our multi-faceted intervention approach. First, engaging physicians directly involved in care delivery through departmental case discussions and individualized feedback has been shown to enhance guideline implementation, consistent with evidence that active educational strategies are more effective than passive approaches for improving clinician practice [22,23].

Second, targeting multiple organizational levels, from emergency department protocols to resident training and nursing practice protocols, addresses complex system barriers to guideline adherence. Implementation science literature demonstrates that multicomponent strategies involving organizational-, provider-, and system-level interventions are associated with improvements in practice change beyond single-component efforts [24]. Third, sustained intervention across multiple cycles over an extended period allows sufficient time for cultural change and avoids regression to baseline practices, an effect seen in longitudinal audit-feedback implementation studies [22].

The persistent challenges regarding blood culture ordering in the emergency department and diagnostic coding accuracy highlight important limitations. These findings suggest that successfully implementing guidelines in secondary care settings requires attention to organizational factors beyond individual provider education, including departmental leadership engagement, administrative reinforcement, and potentially electronic medical record (EMR) decision support [23].

This study has several limitations. First, as a single-center retrospective audit, findings may not be generalizable to tertiary centers or institutions with different staffing structures. Second, disease severity was assessed clinically by the treating physician rather than using a formal, validated scoring tool, which may limit inter-cycle comparability of case-mix severity. Third, the absence of clinical outcome data, such as the length of stay, readmission rates, and pediatric intensive care unit (PICU) transfer, precludes the assessment of whether improved guideline adherence translated into patient benefit. Fourth, attending physician assignment was not captured as a variable, and individual provider variation cannot be excluded as a contributing cofactor. Fifth, granular viral testing data were not consistently documented in the electronic medical records and could not be reliably extracted retrospectively.

Comparison with published literature

Our results are benchmarked against previous quality improvement studies in pediatric bronchiolitis. A systematic review identified that bronchiolitis quality improvement initiatives achieve median 30%-50% reductions in unnecessary investigations and median 40%-60% reductions in bronchodilator use [8]. Our study achieved a 50% reduction in radiography and 75% reduction in salbutamol, representing above-average improvements for multi-cycle audit approaches.

Our findings also align with international experiences implementing bronchiolitis guidelines. Studies from Canada, the United Kingdom, and Australia have demonstrated that regional variation in bronchiolitis management persists despite published guidelines, with similar patterns of radiography overuse and bronchodilator overutilization [2,14]. However, the magnitude of improvement in supportive care measures (particularly nasopharyngeal suctioning) in our study exceeds most published reports, possibly reflecting the sustained engagement of nursing leadership in our quality improvement initiative.

Regional and cultural context

The success of this initiative in a Middle Eastern healthcare system provides important evidence that evidence-based quality improvement methods are broadly applicable across diverse healthcare contexts. Previous concerns that guideline non-adherence in Gulf Cooperation Council (GCC) countries reflected insurmountable cultural or structural barriers appear unfounded, given our sustained improvements across multiple indicators [6]. Implementation challenges often reflect organizational and systemic factors rather than cultural barriers.

## Conclusions

This multi-cycle audit demonstrates that structured, education-driven quality improvement interventions effectively enhance adherence to evidence-based bronchiolitis management guidelines in resource-limited hospital settings. Sustained audit-feedback mechanisms coupled with multidisciplinary collaboration and administrative support enable meaningful practice change. Hospitals should implement similar audit models for other pediatric conditions, particularly within emergency departments. Maintaining improvements requires continued investment in quality improvement infrastructure and electronic clinical decision support. Future research should prospectively evaluate clinical outcomes such as the length of stay, readmission rates, and PICU transfer; assess the long-term sustainability of audit-driven practice change; and conduct multicenter studies across Oman's regional hospital network to validate and scale this quality improvement model.
